# Spatial modelling of agro-ecologically significant grassland species using the INLA-SPDE approach

**DOI:** 10.1038/s41598-023-32077-7

**Published:** 2023-03-27

**Authors:** Andrew Fichera, Rachel King, Jarrod Kath, David Cobon, Kathryn Reardon-Smith

**Affiliations:** 1 School of Mathematics, Physics and Computing, University of Southern Queensland, Toowoomba, 4350 Australia; 2 School of Agriculture and Environmental Science, University of Southern Queensland, Toowoomba, 4350 Australia; 3 Centre for Applied Climate Sciences, University of Southern Queensland, Toowoomba, 4350 Australia

**Keywords:** Agroecology, Climate-change ecology, Ecological modelling

## Abstract

The use of spatially referenced data in agricultural systems modelling has grown in recent decades, however, the use of spatial modelling techniques in agricultural science is limited. In this paper, we test an effective and efficient technique for spatially modelling and analysing agricultural data using Bayesian hierarchical spatial models (BHSM). These models utilise analytical approximations and numerical integration called Integrated Nested Laplace Approximations (INLA). We critically analyse and compare the performance of the INLA and INLA-SPDE (Integrated Nested Laplace Approximation with Stochastic Partial Differential Equation) approaches against the more commonly used generalised linear model (glm), by modelling binary geostatistical species presence/absence data for several agro-ecologically significant Australian grassland species. The INLA-SPDE approach showed excellent predictive performance (ROCAUC 0.9271–0.9623) for all species. Further, the glm approach not accounting for spatial autocorrelation had inconsistent parameter estimates (switching between significantly positive and negative) when the dataset was subsetted and modelled at different scales. In contrast, the INLA-SPDE approach which accounted for spatial autocorrelation had stable parameter estimates. Using approaches which explicitly account for spatial autocorrelation, such as INLA-SPDE, improves model predictive performance and may provide a significant advantage for researchers by reducing the potential for Type I or false-positive errors in inferences about the significance of predictors.

## Introduction

Agriculture is the leading land use in Australia, accounting for 55% of all Australian land use. The largest proportion of this 427 million hectares is identified as areas of grazed native vegetation^1^. Grasslands are, therefore, a vital component of livestock grazing systems, covering 70% of Australia^2^. Previous studies have suggested that grasslands account for 40% of Australia’s gross production value, indicating the significant importance of grasslands to Australia’s agriculture industry^3^. In this context, the ability to more effectively model grassland species distribution is crucial as farmers, researchers and policy-makers adapt to an increasingly variable climate.

Spatial modelling and analysis have gained significant momentum in recent years and have been successfully used in a wide variety of scientific fields. Agricultural systems models are used to solve an extensive variety of problems and support a broad range of decision-making at varying spatial and organisational scales. This highlights the need for a range of reliable and consistent methods for modellers to employ. Many established agricultural systems modelling methods are highly complex and have been developed for specific purposes and crops and require extensive site-level data, which is often unavailable, to appropriately parameterise, narrowing their scope and capability^4^. On the other hand, more generalised statistical approaches, such as the widely used generalised linear model (glm), may have difficulty dealing with complex spatial autocorrelation effects that occur in real-world large-scale agro-ecological datasets and as such incorrectly assess the effects of different environmental factors. There are therefore a variety of important issues, in agricultural systems modelling, highlighting the challenges users face when selecting a modelling approach^5^.

Integrated Nested Laplace Approximations (INLA) have been shown to efficiently and flexibly model complex systems with geographically referenced data^6^. INLA has been widely used in ecological^7,8^ and epidemiological studies^9,10^, but has been little assessed in agricultural settings. The coupling of INLA with the Stochastic Partial Differential Equations (SPDE) approach, allows for the ability to continuously model spatial dependence in the data using a Matern covariance function as a Gaussian Markov Random Field (GMRF)^11^. These approaches offer a more generalisable method for modelling agricultural data while effectively accounting for spatial dependence.

The primary objective of our research is to test the effectiveness of the INLA and INLA-SPDE approaches as alternative or complementary methods for modelling agricultural systems. Using a real-world grassland species dataset, we critically assess the INLA and INLA-SPDE approaches against the established generalised linear model (glm) statistical method using four exemplar grassland species, *Astrebla pectinata*, *Bothriochloa ewartiana*, *Dichanthium fecundum*, and *Themeda triandra*. We test whether an approach which explicitly accounts for spatial autocorrelation (i.e. INLA-SPDE) improves model predictive accuracy and reduces spatial autocorrelation in residuals which can bias model predictions. We present the parameter estimates and spatial predictions that these three approaches exhibit and discuss these differences in the context of the results.

## Results

### Comparison of model performance

**Figure 1:**
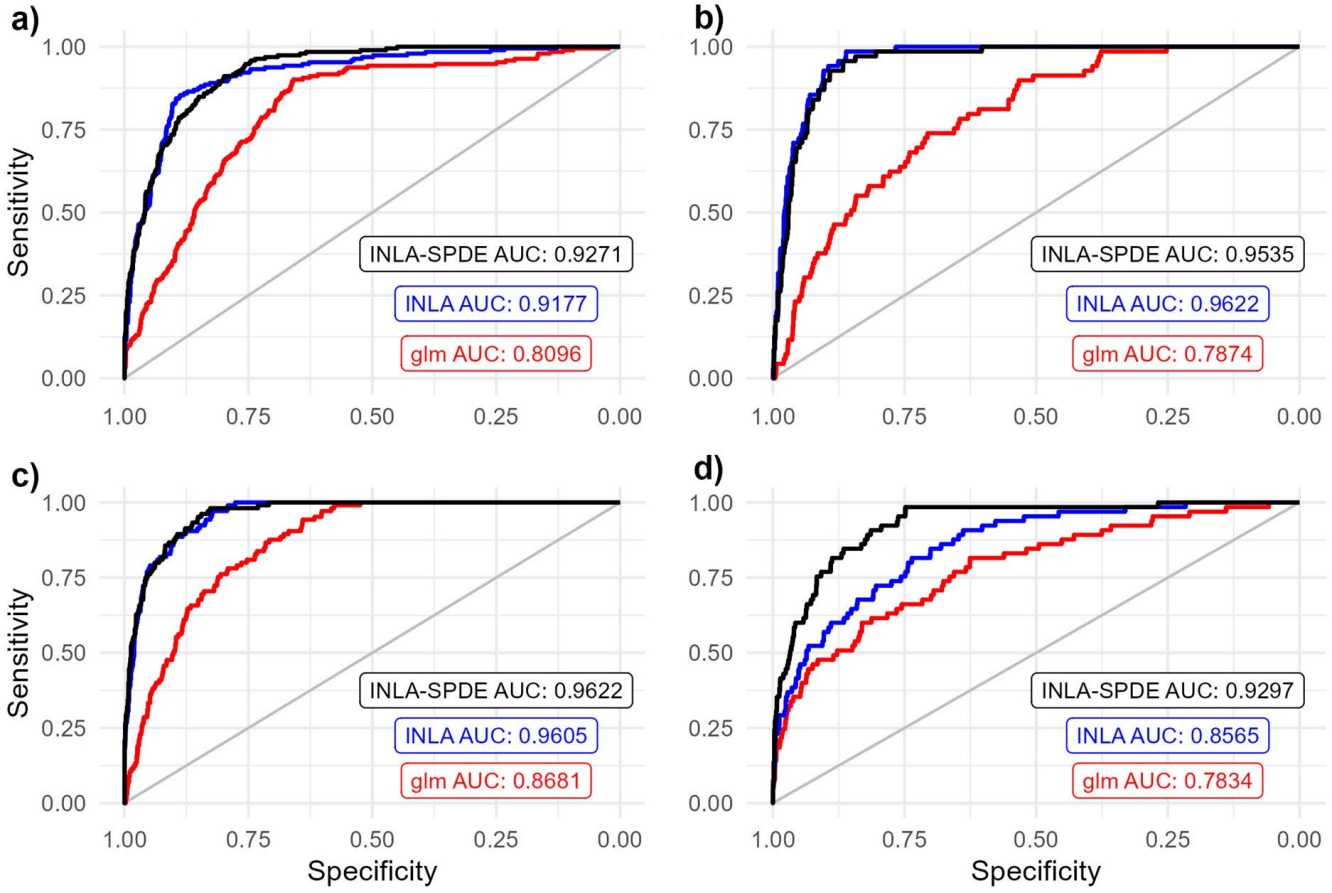
Receiver operator characteristic (ROC) curves and associated Area Under the Curve (AUC) values for the fitted glm models (red), fitted INLA models (blue) and fitted INLA-SPDE models (black) for (**a**) *A. pectinata*, (**b**) *B. ewartiana*, (**c**) *D. fecundum*, and (**d**) *T. triandra*.

The receiver operator characteristic (ROC) curves and area under the curve (AUC) values for the selected grassland species are presented in Fig. 1. For *A. pectinata*, the glm model performed well with an AUC value of 0.8096. The INLA and INLA-SPDE models performed exceptionally with AUC values of 0.9175 and 0.9272 respectively (Fig. 1a). For *B. ewartiana*, the glm model performed acceptably with an AUC value of 0.7874. The INLA and INLA-SPDE models performed better with AUC values of 0.9623 and 0.9535 respectively (Fig. 1b). For *D. fecundum*, the glm model performed well with an AUC value of 0.8681. The INLA and INLA-SPDE models performed better still with AUC values of 0.9606 and 0.9623 respectively (Fig. 1c). For *T. triandra*, the glm model performed acceptably with an AUC value of 0.7834. The INLA model performed well with an AUC value of 0.8565 and the INLA-SPDE model performed exceptionally with an AUC value of 0.9301 (Fig. 1d). For all four grassland species, the INLA and INLA-SPDE models had higher predictive ability compared with the glm approach. The INLA-SPDE approach exhibited excellent predictive ability with AUC values of greater than 0.9 for all four grassland species.

### INLA-SPDE models more adequately account for spatial autocorrelation

The Moran’s I test statistics along with their associated statistical significance for the selected grassland species are presented in Table 1. Moran’s I is a relative measure of the degree of residual spatial autocorrelation remaining after the model has been fitted. For all four species, significant spatial autocorrelation remained in the model residuals using the glm model (*p* < 0.05). For *B. ewartiana*, the INLA model substantially accounted for spatial autocorrelation in the model residuals (*p* > 0.05). However, significant spatial autocorrelation remained in the INLA model residuals for the *A. pectinata*, *D. fecundum* and *T. triandra* species. INLA-SPDE achieved the best results as it was able to effectively account for spatial autocorrelation in the model residuals for the *A. pectinata* and *T. triandra* species. The INLA-SPDE models showed improved ability to account for spatial autocorrelation compared with glm.

**Table 1:** Moran’s I test statistics by species and model type.

Species	glm	INLA	INLA-SPDE
*A. pectinata*	0.0471 (*p* = 0.004)	0.0715 (*p* = 0.011)	0.0433 (*p* = 0.083)
*B. ewartiana*	0.0858 (*p* = 0.001)	0.0263 (*p* = 0.197)	0.0573 (*p* = 0.033)
*D. fecundum*	0.2638 (*p* = 0.000)	0.0889 (*p* = 0.002)	0.0895 (*p* = 0.002)
*T. triandra*	0.1930 (*p* = 0.000)	0.1143 (*p* = 0.000)	0.0142 (*p* = 0.319)

### INLA-SPDE models resulted in different parameter estimates and predictions

**Figure 2:**
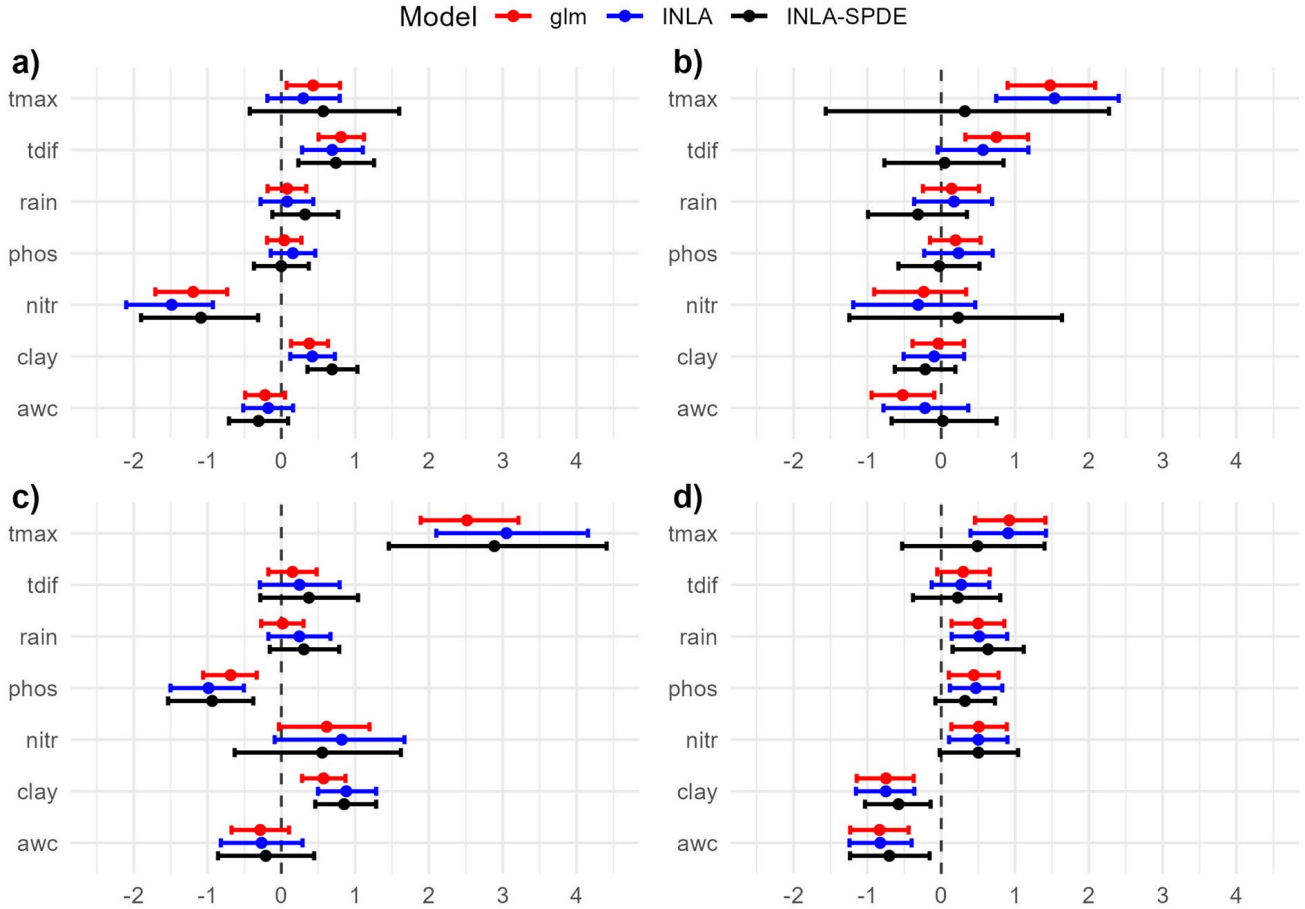
Model parameter estimates (estimates of standardised effect size or model beta coefficients^12^) for climatic and edaphic variables on the probability of species presence based on the global (continent-scale) dataset for (**a**) *A. pectinata*, (**b**) *B. ewartiana*, (**c**) *D. fecundum*, and (**d**) *T. triandra*. Error bars are 95% confidence intervals for glm and 95% credible intervals for INLA and INLA-SPDE. Positive effect sizes indicate that the parameter increases the probability of species presence, while a negative effect indicates the opposite (see Table 2 for variable abbreviations used on the y-axis).

Generally, the results of the INLA-SPDE models showed wider credible intervals than the glm confidence intervals and INLA credible intervals (Fig.  2). Additionally, in some cases INLA-SPDE indicated no associations while the comparative glm and INLA models did.

Figure 2a shows the model parameter estimates (estimates of standardised effect size) for each of the climatic and edaphic variables on the probability of *A. pectinata* species presence. One main difference was observed between the glm, INLA and INLA-SPDE models. The glm model showed that the mean of the maximum temperatures (tmax) was positively associated with higher probabilities of *A. pectinata* species presence while INLA and INLA-SPDE did not indicate an association.

The model parameter estimates of the climatic and edaphic variables predicting *B. ewartiana* species presence are shown in Fig. 2b. A number of differences were found between the three models. The glm and INLA models suggest that the mean of the maximum temperatures (tmax) was positively associated with higher probabilities of *B. ewartiana* species presence. Conversely, INLA-SPDE showed that the mean of the maximum temperatures (tmax) did not have an association with higher probabilities of *B. ewartiana* species presence. The glm model showed that the mean diurnal temperature variation (tdif) was positively associated with higher probabilities of *B. ewartiana* species presence while INLA and INLA-SPDE did not indicate an association. The glm model showed that the available water capacity (awc) was negatively associated with higher probabilities of *B. ewartiana* species presence while INLA and INLA-SPDE did not indicate an association.

Figure 2c shows the climatic and edaphic variable model parameter estimates for the *D. fecundum* species presence. No substantial differences were established between the glm, INLA and INLA-SPDE models for *D. fecundum*. Figure 2d shows the model parameter estimates for each of the climatic and edaphic variables on the probability of *T. triandra* species presence. Three differences between model variables were observed between the glm, INLA and INLA-SPDE models. The glm and INLA models showed that the mean of the maximum temperature (tmax), phosphorus (phos) and nitrogen (nitr) variables were positively associated with higher probabilities of *T. triandra* species presence while INLA-SPDE did not show an association.

Supplementary Figure 1 shows the parameter estimates based on restricted distributions for each species, which allowed us to compare how sensitive parameter estimates were to model scale and data subsetting. INLA-SPDE estimates were largely stable across the global (continent-scale) and localised models (restricted ranges), while the glm and INLA models showed variation in parameter estimates between the two different spatial scales. For example, the maximum temperature (tmax) estimates shifted from a positive association in the global models (Fig. 2b) to a negative association based on the localised (Supplementary Figure 1b) for the glm and INLA models.

**Figure 3:**
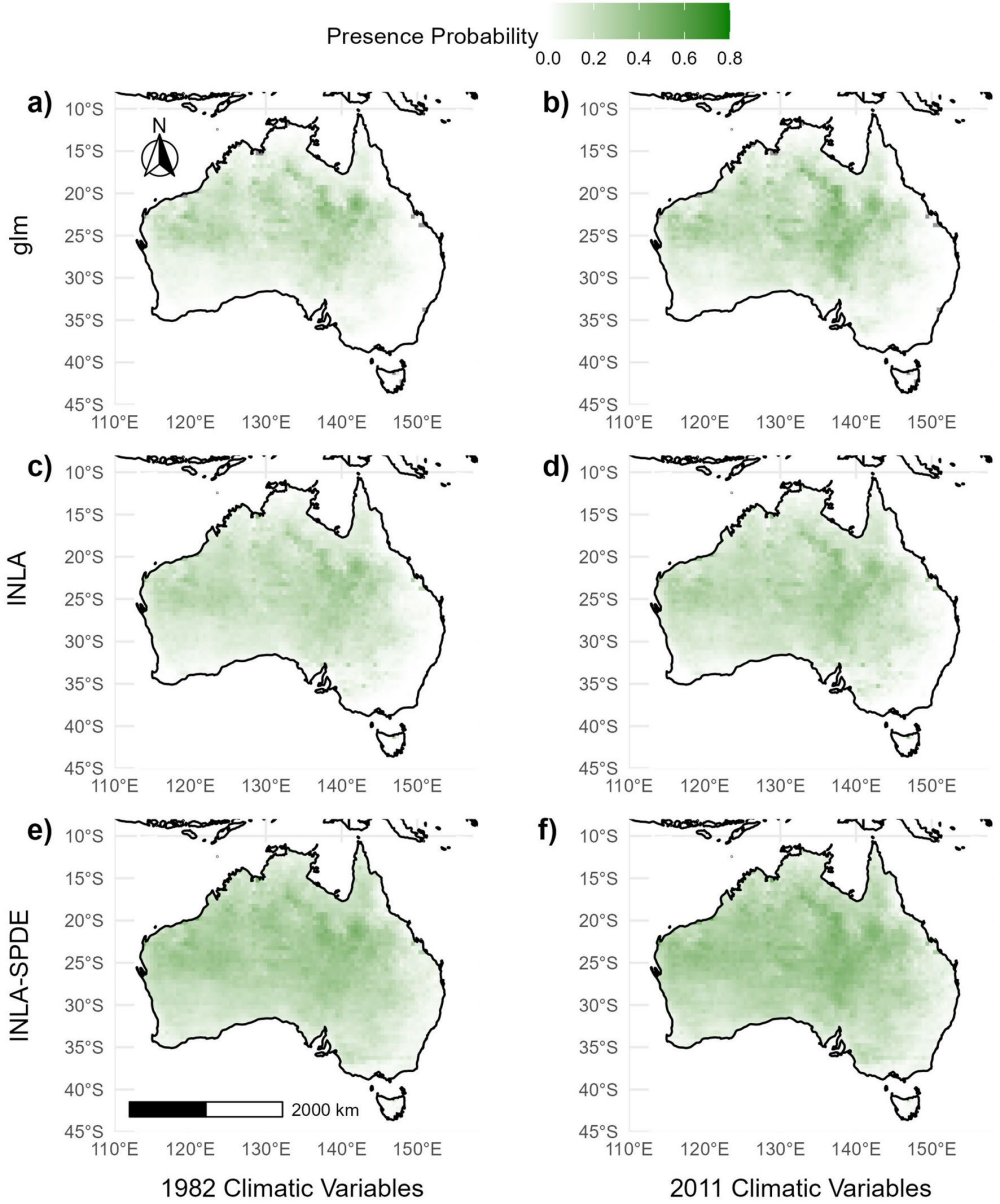
Probabilistic spatial extrapolation plots of (**a**) *A. pectinata* species presence modelled using climatic data from 1982 using glm, (**b**) *A. pectinata* species presence modelled using climatic data from 2011 using glm, (**c**) *A. pectinata* species presence modelled using climatic data from 1982 using INLA, (**d**) *A. pectinata* species presence modelled using climatic data from 2011 using INLA, (**e**) *A. pectinata* species presence modelled using climatic data from 1982 using INLA-SPDE and (**f**) *A. pectinata* species presence modelled using climatic data from 2011 using INLA-SPDE. For spatial extrapolation plots of *B. ewartiana*, *D. fecundum*, and *T. triandra*, refer to Supplementary Figures 3, 4 and 5.

Figure 3 shows the spatial extrapolation plots of *A. pectinata* species presence modelled using the glm, INLA and INLA-SPDE approaches with historical climatic data from 1982 (a hot and dry year) and 2011 (a cold and wet year). All three models showed higher probabilities of *A. pectinata* species presence in central-western Queensland, north-eastern South Australia, and south-eastern Northern Territory in both climatic extremes. These predictions align well with historical *A. pectinata* species observations^13^.

The glm and INLA models showed no species presence in south-eastern Queensland, eastern New South Wales, eastern Victoria and Tasmania which is supported by historical observations of *A. pectinata*^13^. Conversely, INLA-SPDE predicted very low probabilities (between 5 and 10%) of *A. pectinata* species presence in these areas. The *A. pectinata* species presence response was higher (typically between 0 and 4%) for all three models using the 2011 climatic variables (cold and wet) than when using the 1982 climatic variables (hot and dry).

## Discussion

In this paper, we demonstrated an effective and efficient technique for spatially modelling and analysing agricultural data using Bayesian hierarchical spatial models (BHSM). Ignoring spatial auto-correlation increases the possibility of biased parameter estimates and overly optimistic standard errors which can lead to misleadingly narrow confidence and credible intervals^14^ and erroneous inferences about variable importance and reduced predictive performance.

We critically assessed and compared the INLA and INLA-SPDE approaches against the established generalised linear model (glm) statistical method using four exemplar grassland species. We show the higher predictive ability of the INLA and INLA-SPDE approaches using ROC and AUC values. We also assessed the ability of these models to better account for spatial autocorrelation in the model residuals. Finally, we presented the differences in the model parameter estimates and predictions between the three modelling approaches.

All three modelling approaches easily handled modelling spatially diverse grassland species distribution data. The INLA and INLA-SPDE approaches exhibited higher predictive ability over the glm approach for all four exemplar grassland species. However, the INLA-SPDE approach showed the best predictive ability with AUC values of greater than 0.9 in all cases. INLA-SPDE’s higher predictive ability over the INLA and glm approaches was exemplified when classifying *T. triandra* species presence/absence. The higher performance of INLA-SPDE for the *A. pectinata*, *D. fecundum* and *T. triandra* species could simply be down to specific spatial structures prevalent in those two species that are more suited to INLA-SPDE. Our results are similar to previous studies utilising INLA-SPDE in the context of ecological species distribution modelling^15,16^.

This improvement in predictive ability is likely due to the INLA-SPDE approach appropriately modelling the spatial variation through the use of an explicit Matern spatial dependence structure. While the climatic and edaphic predictors inherently possess spatial dependence (‘macro-scale’ spatial dependence) that is captured by fitting the main effects in each of the models, the difference between the INLA and the INLA-SPDE models is in how the remaining spatial dependence in the residuals (‘micro-scale’ spatial dependence) is captured by the different spatial dependence structures. In the INLA-SPDE models the Matern spatial dependence structure allows the micro-scale spatial variation to be continuously modelled across the spatial range (continent-wide), providing a more precise representation of the spatial variability, including any localised clustering.

The INLA-SPDE model parameter estimates produced wider intervals than glm and INLA models in all cases. Previous studies using Bayesian spatial models have produced similar results^17^. The large INLA-SPDE credible interval for *B. ewartiana* indicating no association between maximum temperature (tmax) and the response variable is notably very different to the narrower INLA and glm intervals indicating a positive association (Fig. 2b). Additionally, the parameter estimates were stable when comparing the global versus localised distribution models for the INLA-SPDE, but not for the glm and INLA. Therefore, the INLA-SPDE approach which models the spatial dependence continuously may provide an advantage for researchers, reducing the potential for false-positive errors in inferences about the importance and direction of effects.

In our study, the glm model not accounting for spatial autocorrelation may be potentially incorrectly inferring that higher maximum temperatures (tmax) are positively related to the presence of several grassland species (i.e. a false-positive error). The positive maximum temperature parameter estimates from the glm are also inconsistent with previous research. In general hotter and drier climates are likely to negatively affect most grassland species^18^. Previous studies on our focus species also suggest that a positive relationship between higher temperatures and grassland species occurrence should be interpreted with caution. Studies on *T. triandra* in Southern Africa, have shown little difference in germination rates between 15 and 35 °C^19^. For species in the Astrebla genus, positive growth responses to temperature have been found, but only up to maximum temperatures of 30 °C and with optima at 28 °C^20^, which is well below the 35 °C temperatures that our data extends to.

The importance of deriving accurate and unbiased estimates of the effects of environmental factors, and especially climatic stressors, on the distribution of species has significant implications for agro-ecological research^21^. Our findings suggest that the use of a glm, which does not account for spatial autocorrelation, could incorrectly lead to the conclusion that higher maximum temperatures are beneficial to three of the four grassland species we modelled. In contrast, the INLA-SPDE approach that accounted for complex spatial autocorrelation patterns found that there was no such positive relationship between maximum temperatures and the occurrence of grassland species. In the context of climate change incorrectly ascribing a positive effect to a climatic factor could lead to inaccurate assessments of climate risk and in turn undermine adaptation strategies.

The approach we employed in this research only utilised linear additive effects for all three models to improve computation time. However, previous studies have shown that the use of linear relationships when using the INLA-SPDE approach may produce less precise results^15^. Although all models performed well, the use of non-linear effects would likely add some improvement to the obtained results, but at the expense of computation time. The glm model was computationally less expensive than both of the INLA and INLA-SPDE approaches. With our dataset, model fitting using a glm took less than a second, fitting with INLA took 7 s and fitting with INLA-SPDE took 82 s when fitting for *A. pectinata*. Previous findings have shown that for a large dataset (over 260,000 observations), a generalised additive model (GAM) ran in a few minutes whereas the INLA models took hours^7,15^. The choice between precision and computation time is study dependent and should be decided based on the objectives and desired outcomes of the researcher.

Spatial extrapolation plots generated from the INLA-SPDE models predicted very low probabilities (between 5 and 10%) of *A. pectinata* species presence in areas where there were no historical observations. Spatially explicit models, such as INLA-SPDE, are designed to capture the micro-scale spatial effect inherent in all geographically referenced data. The differences in the predicted species presence in areas with no historical observations is likely due to the difference in the estimated practical range when using INLA-SPDE (compared with a conventional semi-variogram in Supplementary Table 2; Supplementary Figures 6, 7). The practical range is the distance at which geographically referenced observations are no longer correlated. INLA-SDPE estimates that the range where correlations level off is far greater than when using a glm. The spatial variability in an agro-ecological system ultimately affects the relationships between species and their environments^22^, highlighting the importance in selecting an appropriate modelling approach.

In this study, the INLA and INLA-SPDE approaches were evaluated using four selected exemplar grassland species as a way to demonstrate their effectiveness in an agro-ecological context. Although the use of INLA in this context is rare^23–25^, previous applications have been shown to provide reliable results. Evaluating the performance of INLA and INLA-SPDE using several types of agricultural data or simulated agricultural data would provide further opportunities to test the effectiveness of these methods. In order to further validate the results obtained in this study, there is potential for cross-validation techniques to be utilised to compare the predictive power of the INLA-SPDE models when predicting species presence at unobserved locations^26^. Further research is needed to understand where it is appropriate to use the computationally less expensive INLA with a pseudo-location random effect approach or to utilise the spatially explicit INLA-SPDE approach. A simulated species distribution dataset could be utilised to test the boundaries where the INLA-SPDE approach is necessitated.

## Methods

### Grassland species data

**Figure 4:**
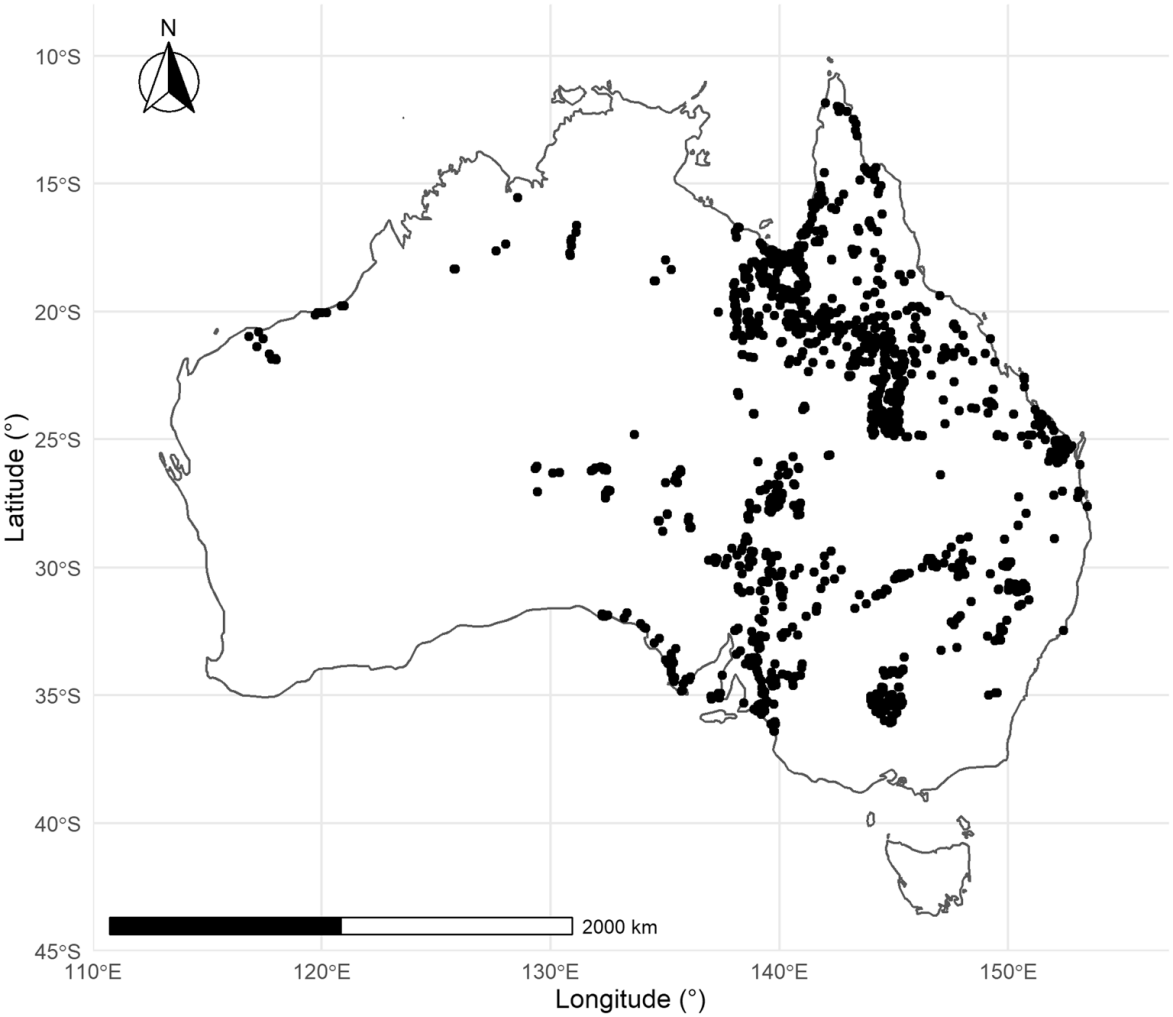
Australia with the sample site locations shown in black.

Grassland species data was obtained from the Australian Ecological Knowledge and Observation System (AEKOS) which is an open-source Terrestrial Ecosystem Research Network (TERN) eco-informatics data portal containing species cover data from across Australia^27^. The dataset consists of binary species distribution data identifying species presence and absence. The dataset was subset to include all observations occurring in grassland systems (labelled in the AEKOS as ’Grasslands’ or ’Swampy Grasslands’). All data cleaning, preparation and analyses were performed using *R* software^28^ and *RStudio*^29^. Four agro-ecologically significant grassland species were selected in this study. *A. pectinata* (n = 192), *B. ewartiana* (n = 69), *D. fecundum* (n = 105), and *T. triandra* (n = 65) are all agriculturally significant perennial grasses that are highly palatable fodder for natively grazed livestock^30–33^. Figure 4 shows all sample locations from the AEKOS portal for the four exemplar species.

### Climatic and edaphic data

Four climatic variables and five edaphic variables were extracted using *R* scripts. The climatic variables were extracted from the Australian Gridded Climate Data (AGCD), which provides high-quality historical and ongoing real time climate analyses for Australia at a 5 km resolution^34^. The edaphic data was sourced from the Commonwealth Scientific and Industrial Research Organisation’s (CSIRO) Soil and Landscape Grid of Australia which provides modelled soil characteristics at a spatial resolution of 3 arc-s (approximately 90 m)^35^. The climatic and edaphic variable values used in our models were extracted at the same locations as the observed species’ presence/absence locations.

### Climatic and edaphic variables

For each species, the following variables were obtained: available water capacity (awc; %), mass fraction of carbon by weight in the < 2 mm soil material (carb; %), mass fraction of soil < 2 $\upmu$m from the < 2 mm soil material profile (clay; %), mass fraction of total nitrogen in the soil by weight (nitr; %), mass fraction of total phosphorus in the soil by weight (phos; %), cumulative rainfall 12 months prior to the sample observation date (rain; mm), mean of the maximum temperatures at the sample site 12 months prior to the sample observation date (tmax; $^\circ$C), mean of the minimum temperatures at the sample site 12 months prior to the sample observation date (tmin; $^\circ$C), and the difference between tmax and tmin at the sample site (tdif; $^\circ$C).

Outliers were removed using the interquartile range criterion, which refers to observations that fall outside of $\pm 1.5 \times$ IQR^36^. In order to avoid the issues associated with multicollinearity, Pearson’s correlation coefficient (*r*) was calculated for all pairs of covariates prior to model fitting using the corrplot package in R^37^. Pairs of covariates with Pearson correlation coefficients $|r| > 0.7$ were identified and one of the variables removed prior to model fitting^38^. The variables carb and tmin were identified through this process and removed (Supplementary Figure 2). Table 2 shows the summary statistics for each of the variables.

**Table 2:** Summary statistics of the climatic and edaphic variables used in the study.

Variable abbr.	Variable name	Units	Minimum	Mean	Maximum	Spatial resolution
Awc	Available water capacity	%	9.85	15.13	19.84	3 arc-s
Clay	Clay content in soil	%	2.90	28.88	57.66	3 arc-s
Nitr	Nitrogen content in soil	%	0.02	0.06	0.15	3 arc-s
Phos	Phosphorus content in soil	%	0.01	0.03	0.05	3 arc-s
Rain	Cumulative rainfall$^{\text {a}}$	mm	4.60	511.07	1468.68	5 km
tdif	Mean diurnal temperature variation$^{\text {a}}$	$^\circ$C	8.23	13.69	18.30	5 km
tmax	Mean maximum temperature$^{\text {a}}$	$^\circ$C	18.66	29.02	35.45	5 km

$^{\text {a}}$12 months prior to species presence/absence observation.

### Analyses

The probability of species presence was quantified utilising three differing modelling strategies, a generalised linear model (glm) a Bayesian hierarchical linear mixed model (INLA) and a Bayesian hierarchical spatial model (INLA-SPDE). All three models included climatic and edaphic variables as linear fixed effects, with the INLA and INLA-SPDE models including a random spatial effect to account for potential spatial autocorrelation in the data.

The glm model was fit for each species as follows:

1 $$\begin{aligned} g(\mu _i) = \alpha + \beta _1X_{1i} + \beta _2X_{2i} + \beta _3X_{3i} + \beta _4X_{4i} + \beta _5X_{5i} + \beta _6X_{6i} + \beta _7X_{7i} \end{aligned}$$

where *g*() is the logit link function with binomial family, $\mu _i$ is the mean response (probability of species presence), $\alpha$ is the intercept, $\beta _kX_{ki}$ are the covariate parameters and covariates (1: nitr, 2: awc, 3: phos, 4: clay, 5: rain, 6: tmax, and 7: tdif), and $i = 1,\ldots , n$ are the sample site observations.

The INLA and INLA-SPDE models were implemented using the R-INLA package in R^6^. The INLA model was fit for each species as follows:

2 $$\begin{aligned} g(\mu _i) = \alpha + \beta _1X_{1i} + \beta _2X_{2i} + \beta _3X_{3i} + \beta _4X_{4i} + \beta _5X_{5i} + \beta _6X_{6i} + \beta _7X_{7i} + f_s(X_{si}) \end{aligned}$$

where *g*() is the logit link function with binomial family, $\mu _i$ is the mean response (probability of species presence), $\alpha$ is the intercept, $\beta _kX_{ki}$ are the covariate parameters and covariates (1: nitr, 2: awc, 3: phos, 4: clay, 5: rain, 6: tmax, and 7: tdif), $f_s(X_{si})$ is a spatial error term, and $i = 1,\ldots , n$ are the sample site observations.

The spatial error term in the INLA model, $f_s(X_{si})$, was constructed by clustering the sample sites using a mean shift clustering algorithm from the LPCM package in R^39^. The mean shift used 200 iterations with 10% of the data range adopted for the bandwidth. Each cluster identified was assigned a different ID, $f_s(X_{si}) =$1, 2, 3, ..., n, where n is the number of clusters identified. The clusters represented spatially grouped sites and acted as a pseudo-location term.

The INLA-SPDE model was fit for each species as follows:

3 $$\begin{aligned} g(\mu _i) = \alpha + \beta _1X_{1i} + \beta _2X_{2i} + \beta _3X_{3i} + \beta _4X_{4i} + \beta _5X_{5i} + \beta _6X_{6i} + \beta _7X_{7i} + {\varvec{X}} \end{aligned}$$

where *g*() is the logit link function with binomial family, $\mu _i$ is the mean response (probability of species presence), $\alpha$ is the intercept, $\beta _kX_{ki}$ are the covariate parameters and covariates (1: nitr, 2: awc, 3: phos, 4: clay, 5: rain, 6: tmax, and 7: tdif), ${\varvec{X}}$ is the latent Gaussian field, and $i = 1,\ldots , n$ are the sample site observations.

Under the INLA-SPDE framework, we consider the random field ${\varvec{X}}$ to be normally distributed with a mean of 0 and the joint covariance defined as a Matern covariance structure as follows:

4 $$\begin{aligned} r({\textbf {u}}, {\textbf {v}}) = \frac{\sigma ^2}{2^{\nu -1}\Gamma (\nu )}(\kappa ||{\textbf {v}}-{\textbf {u}}||^{\nu }K_\nu (\kappa ||{\textbf {v}}-{\textbf {u}}||) \end{aligned}$$

where $K_\nu$ is the modified Bessel function, $\nu > 0$ and $\kappa > 0$ are scaling parameters, $\sigma ^2$ is the marginal variance, $\Gamma (\cdot )$ is the gamma function and $||{\textbf {v}}-{\textbf {u}}||$ indicates the Euclidean distance between points **v** and **u**^40^.

The INLA-SPDE approach incorporates the Matern covariance structure by constructing a non-convex spatial mesh using the inla.mesh.2d and inla.spde2.matern functions in R^6^. The Matern covariance structure is a solution to the INLA-SPDE’s form first presented by Lindgren, Rue, and Lindstrom^40^. Other covariance functions can be used to approximate a Gaussian Markov Random Field, as demonstrated by Rue and Tjelmeland^41^, however these other covariance structures could only be computed on a regular grid. The R-INLA package utilises a Matern covariance structure which allows irregularly spaced triangulated data. We specified the triangulation edge sizes within the inla.mesh.2d function. A number of mesh configurations were constructed using the inla.spde2.matern function. Each of the constructed meshes were tested and compared using ROC AUC, computation time and Moran’s I to inform the most appropriate configuration to adopt (Supplementary Figure 8; Supplementary Table 1). The mesh configuration which produced the lowest Moran’s I test statistic (best accounted for spatial autocorrelation) was selected for the INLA-SPDE models. The 5,10 mesh configuration produced the lowest Moran’s I test statistic (I = 0.0440, *p* = 0.08). A projection matrix was produced using the inla.spde.make.A function along with the observed and prediction data stacks using the inla.stack function in R^6^. The model formula was specified and the model was fit using the inla function in R^6^.

### Model performance and residual spatial autocorrelation

The predictive ability of the three modelling approaches was measured using ROC plots (Fig. 1), which compare a model’s specificity against it’s sensitivity. Lines above the diagonal 1:1 line represent performance where the true positive rate is higher than the false positive rate. A model’s specificity is defined as the number of true negative responses divided by the sum of true negative and false positive responses given a certain threshold. Conversely, a model’s sensitivity is defined as the number of true positive responses divided by the sum of true positive responses and false negative responses given the same certain threshold. The ROC curve is produced by sweeping the threshold between 0 and 1 and plotting the resulting specificity and sensitivity. AUC values indicating the area under the ROC curves were also calculated with values closest to 1 indicating the best performance^42^. AUC values between 0.7 and 0.8 were considered acceptable, 0.8 and 0.9 were considered excellent and more than 0.9 was considered outstanding^43^.

Plots of the standardised parameter estimates for the climatic and edaphic variables for each of the three models and four species were created (Fig. 2). As is common practice when displaying and interpreting parameter estimates for each of these model types, 95% confidence intervals were calculated for the glm estimates and 95% credible intervals were calculated for the INLA and INLA-SPDE estimates. While derived under distinctly different frameworks (frequentist and Bayesian respectively), both interval types are used in practice to summarise the uncertainty related to the unknown population mean parameter values being estimated.

Additionally, we tested the stability of the parameter estimates by sub-setting the data. We did this by restricting the datasets to the latitude and longitude ranges of the presences for each species. Where parameter estimates are inconsistent at the global (continent-wide) and localised scale, this could indicate that the global scale model is biased and subsequent inference inappropriate for spatial extrapolation^44^ (Supplementary Figure 1).

Spatial extrapolation plots presented the mean probability of species presence (fitted model responses) at unobserved locations for each of the three modelling approaches. The spatial extrapolation plots were constructed using the sf^45^, ggspatial^46^, and ggplot2^47^ packages in R. The posterior distributions of the estimated practical ranges were calculated using the inla package in R, utilising the inla and inla.spde2.result functions.These functions interpolate using a basis function representation with random Gaussian weights^6^.

### Methods summary

**Figure 5:**
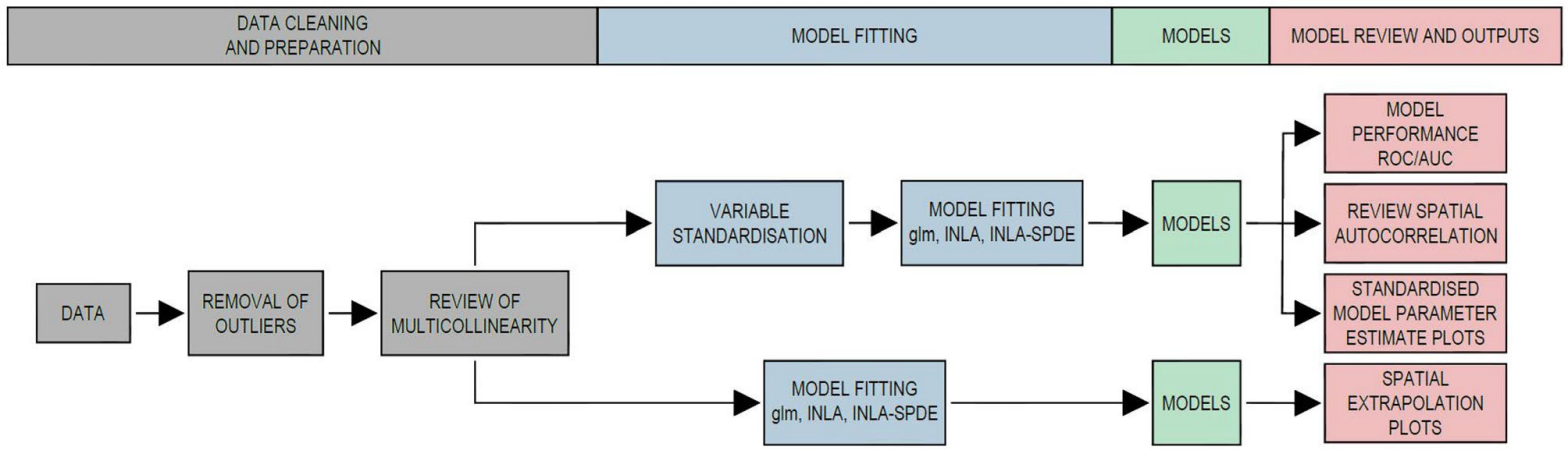
Schematic flowchart of the overall spatial modelling approaches utilised.

Figure 5 shows a schematic flowchart of the overall spatial modelling approaches adopted in this study. The variable standardisation flow allows for the comparison of the effect sizes of individual model parameters against each other on the same scale for all three models. The non-standardisation flow was used for the spatial extrapolation plots only.

## Supplementary Information

Supplementary Information.

## Data Availability

The analyses are based on publicly available datasets. Grasslands species data is from http://www.portal.aekos.org.au/. Climatic data is from http://www.bom.gov.au/climate/data/. Edaphic data is from https://data.csiro.au/collection/csiro:10168v5. The final cleaned dataset linking the climatic and edaphic data to species presence for analyses is stored at Harvard dataverse https://doi.org/10.7910/DVN/WB3AMO.
