# Supplementary material for: Spatial modelling of agro-ecologically significant grassland species using the INLA-SPDE approach
Source: Sci Rep. 2023 Mar 27;13:4972. doi: 10.1038/s41598-023-32077-7 (PMC10043286; doi:10.1038/s41598-023-32077-7)
Supplement: Supplementary file 1 — Supplementary Information. [file 41598_2023_32077_MOESM1_ESM.pdf]

Supplementary information

Title: Spatial modelling of agro-ecologically significant grassland species using the INLA-SPDE approach

[1]Andrew Fichera [1,*]Rachel King [2,3]Jarrod Kath [3]David Cobon [2,3]Kathryn Reardon-Smith
[1]University of Southern Queensland, School of Mathematics, Physics and Computing, Toowoomba, 4350, Australia
[2]University of Southern Queensland, School of Agriculture and Environmental Science, Toowoomba, 4350, Australia
[3]University of Southern Queensland, Centre for Applied Climate Sciences, Toowoomba, 4350, Australia

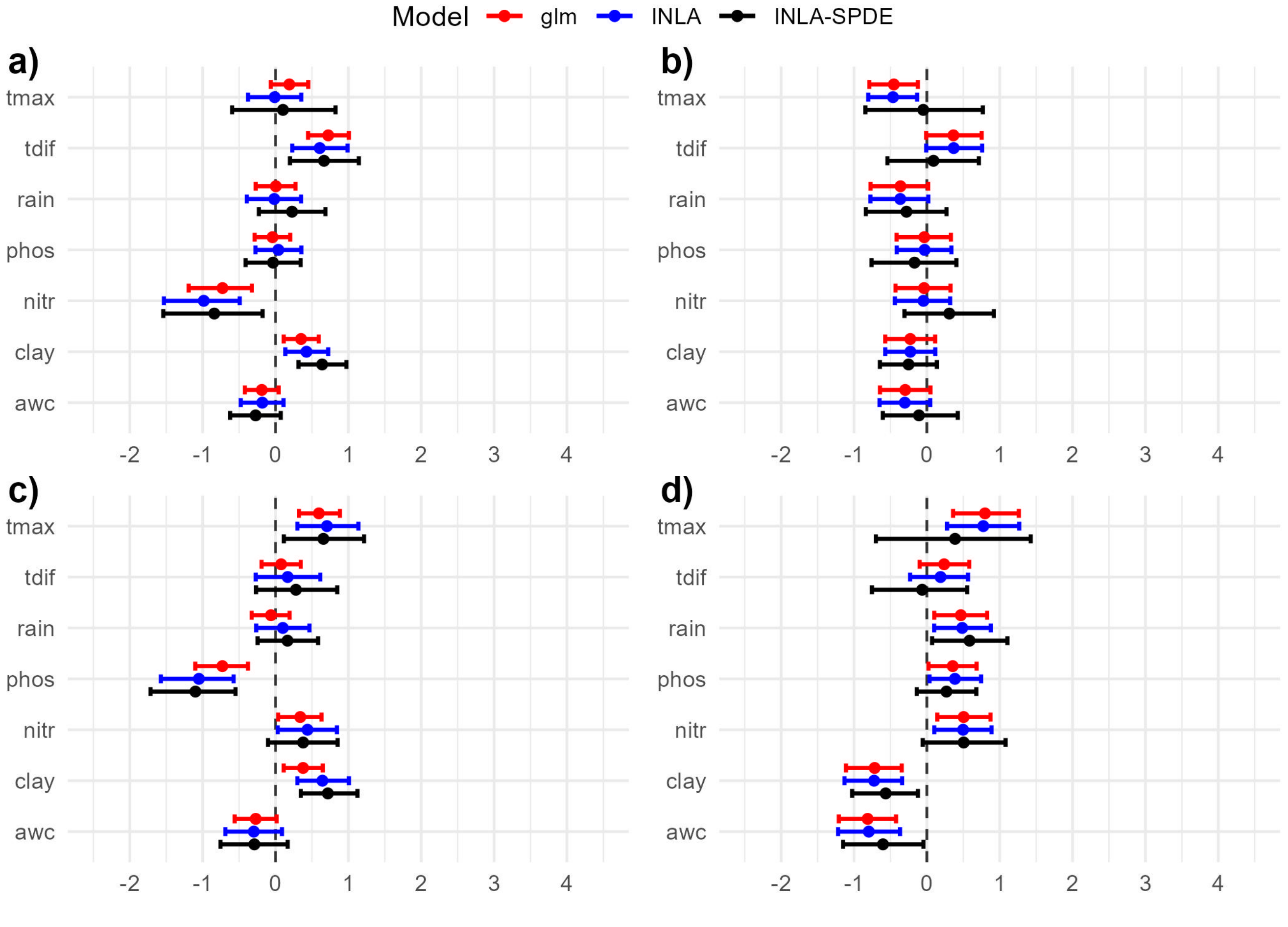

Supplementary Figure 1. Model parameter estimates (estimates of standardised effect size or model beta coefficients[12]) for climatic and edaphic variables on the probability of species presence based on the subset dataset for a) *A. pectinata*, b) *B. ewartiana*, c) *D. fecundum*, and d) *T. triandra*. Error bars are 95% confidence intervals for glm and 95% credible intervals for INLA and INLA-SPDE. Positive effect sizes indicate that the parameter increases the probability of species presence, while a negative effect indicates the opposite. (See Table 2 for variable abbreviations used on the y-axis).

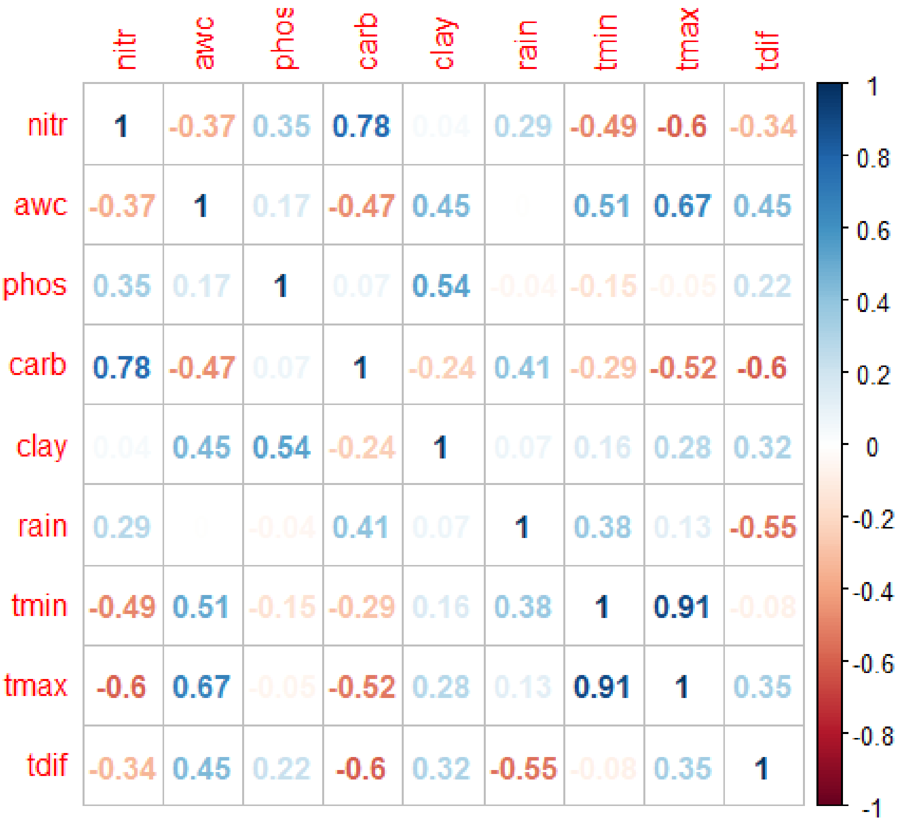

Supplementary Figure 2. Correlation matrix between model covariates. Positive correlations are shown in blue, negative correlations are shown in red. Stronger correlations are shown brighter, weaker correlations are shown fainter.

Supplementary Table 1. INLA-SPDE spatial mesh configurations using maximum triangulation size arguments. The first value in the argument pair is the maximum triangle size in the inner domain and the second value is the maximum triangle size in the outer domain.

Triangulation Edge Sizes	ROCAUC	Computation Time (sec)	Moran's I	*p* value
10,10	0.9428	21.7	0.0505	$p = 0.053$
7,10	0.9428	24.6	0.0506	$p = 0.053$
5,10	0.9270	20.8	0.0440	$p = 0.080$
2,4	0.9268	18.2	0.0446	$p = 0.077$
1,10	0.9261	18.9	0.0447	$p = 0.074$
1,2	0.9258	21.2	0.0455	$p = 0.073$

Supplementary Table 2. Differences in estimated practical range between semi-variogram and INLA-SPDE.

Species	Semi-variogram Range	INLA-SPDE Range
A. pectinata	1.68°	3.17°
B. ewartiana	0.65°	5.88°
D. fecundum	1.16°	1.47°
T. triandra	0.45°	3.52°

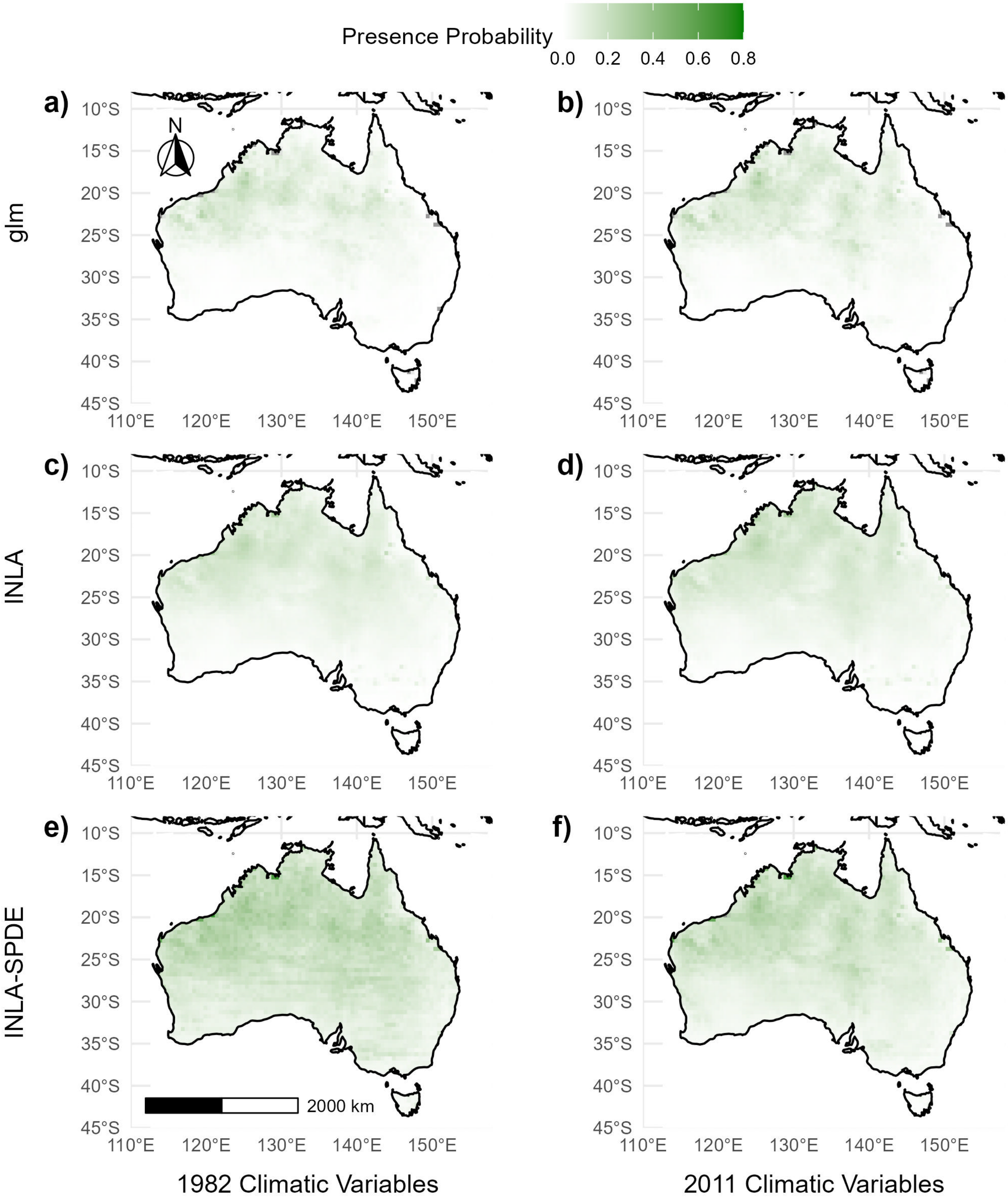

Supplementary Figure 3. Probabilistic spatial extrapolation plots of a) *B. ewartiana* species presence modelled using climatic data from 1982 using glm, b) *B. ewartiana* species presence modelled using climatic data from 2011 using glm, c) *B. ewartiana* species presence modelled using climatic data from 1982 using INLA, d) *B. ewartiana* species presence modelled using climatic data from 2011 using INLA, e) *B. ewartiana* species presence modelled using climatic data from 1982 using INLA-SPDE and f) *B. ewartiana* species presence modelled using climatic data from 2011 using INLA-SPDE.

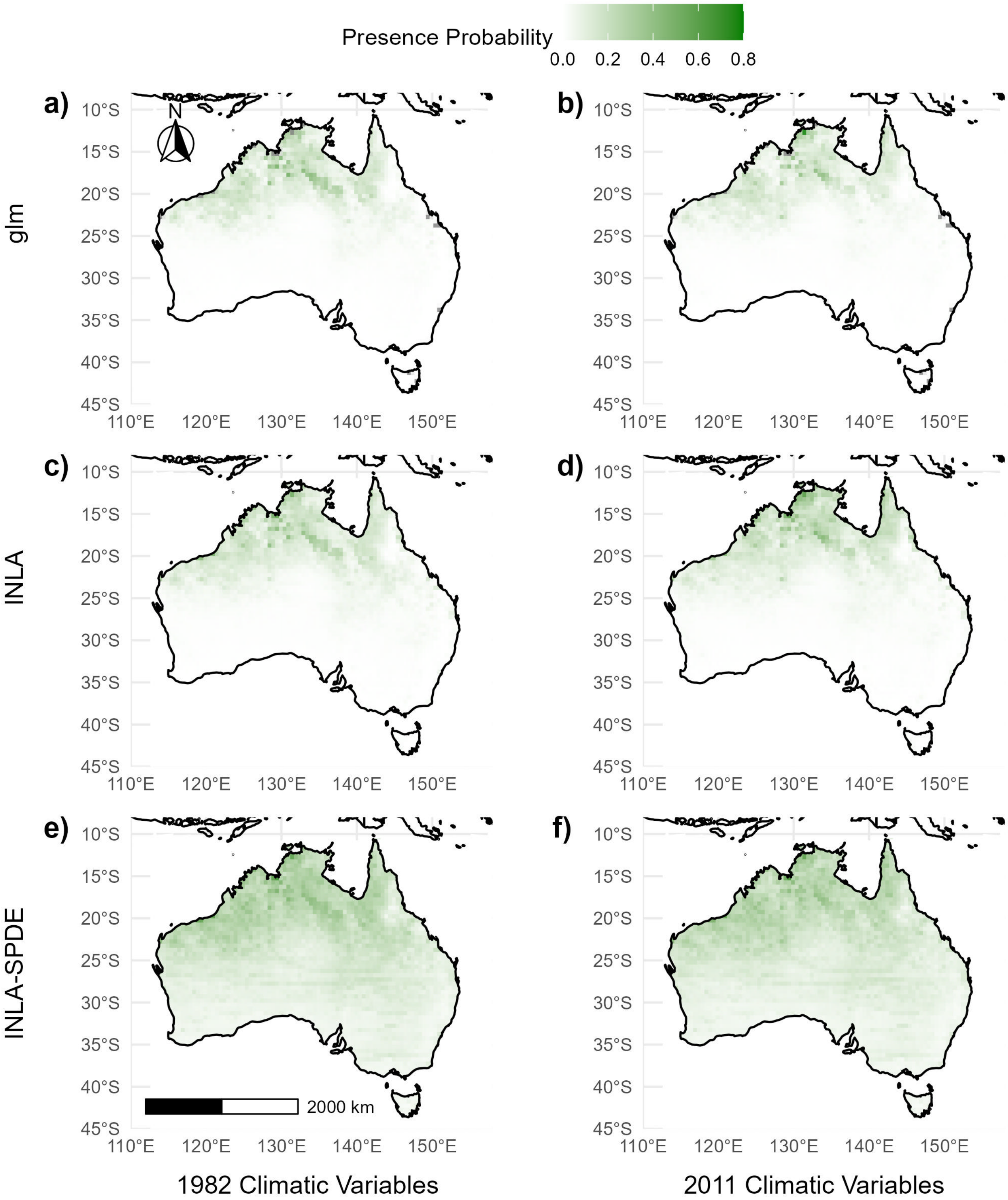

Supplementary Figure 4. Probabilistic spatial extrapolation plots of a) *D. fecundum* species presence modelled using climatic data from 1982 using glm, b) *D. fecundum* species presence modelled using climatic data from 2011 using glm, c) *D. fecundum* species presence modelled using climatic data from 1982 using INLA, d) *D. fecundum* species presence modelled using climatic data from 2011 using INLA, e) *D. fecundum* species presence modelled using climatic data from 1982 using INLA-SPDE and f) *D. fecundum* species presence modelled using climatic data from 2011 using INLA-SPDE.

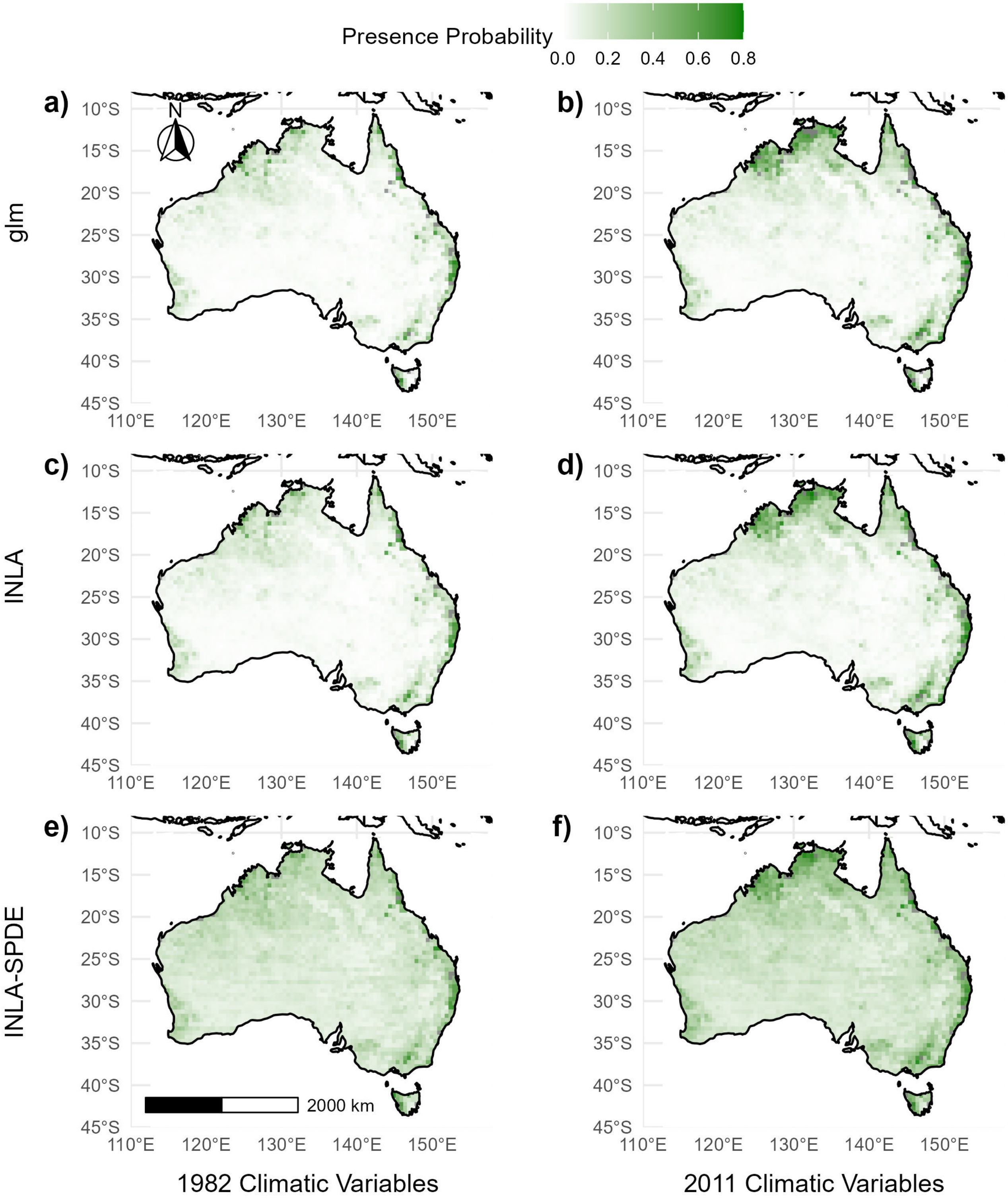

Supplementary Figure 5. Probabilistic spatial extrapolation plots of a) *T. triandra* species presence modelled using climatic data from 1982 using glm, b) *T. triandra* species presence modelled using climatic data from 2011 using glm, c) *T. triandra* species presence modelled using climatic data from 1982 using INLA, d) *T. triandra* species presence modelled using climatic data from 2011 using INLA, e) *T. triandra* species presence modelled using climatic data from 1982 using INLA-SPDE and f) *T. triandra* species presence modelled using climatic data from 2011 using INLA-SPDE.

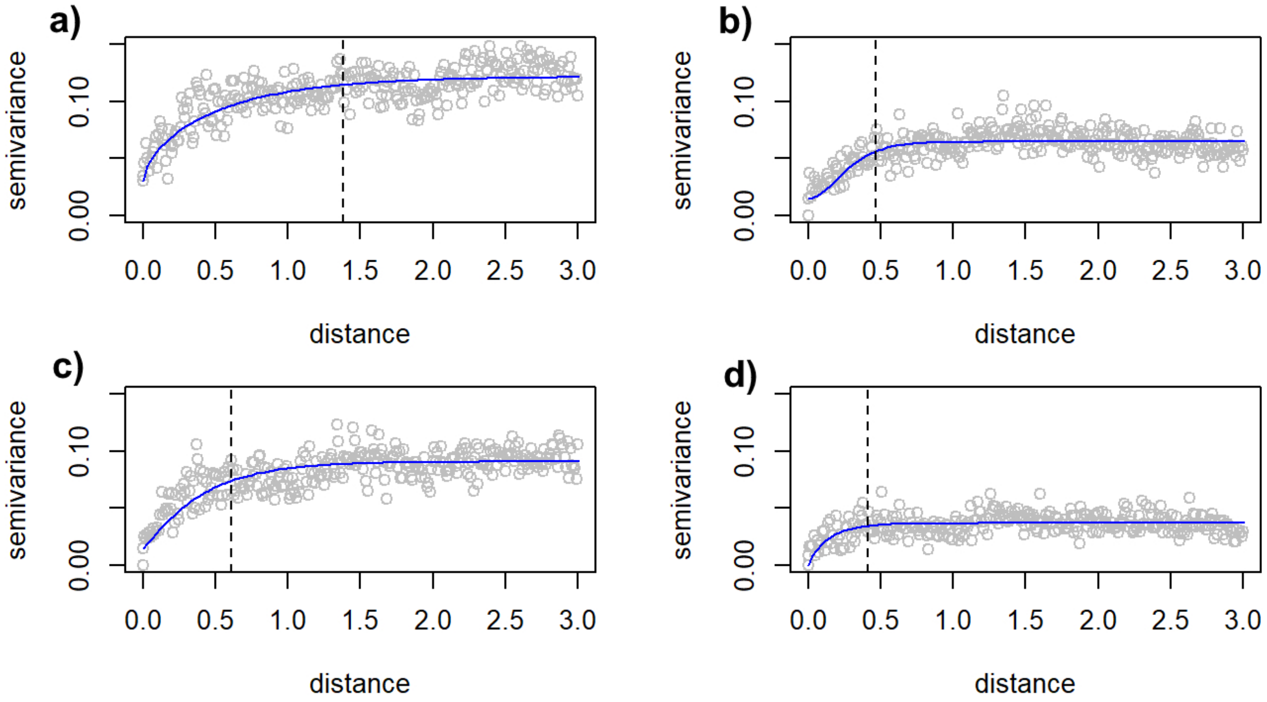

Supplementary Figure 6. Semi-variograms of a) *A. pectinata*, b) *B. ewartiana*, c) *D. fecundum*, and d) *T. triandra*, where distance is measured in arc degrees, showing estimated Practical range (black dashed line), and fitted Matern covariance structure (blue solid line).

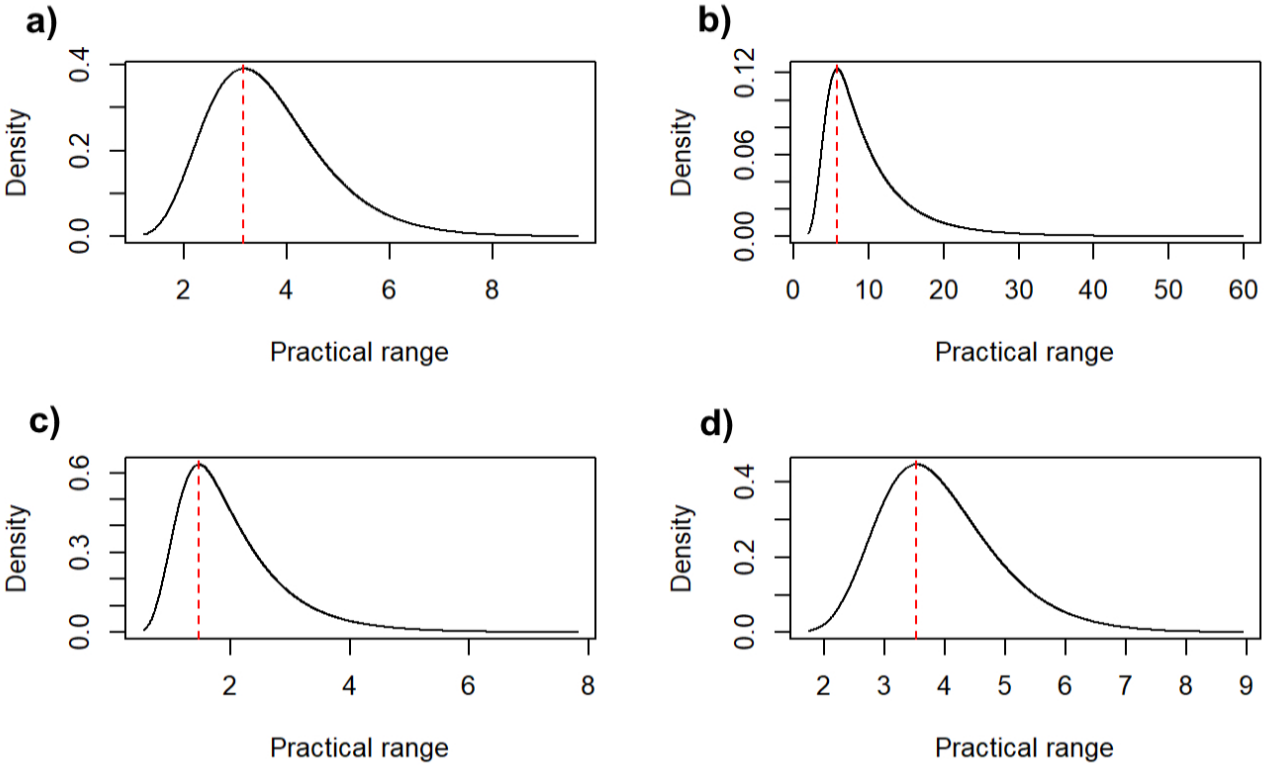

Supplementary Figure 7. Posterior distributions of estimated practical range for a) *A. pectinata*, b) *B. ewartiana*, c) *D. fecundum*, and d) *T. triandra* using INLA-SPDE, where Practical range is measured in arc degrees, showing estimated Practical range density (black solid line), and maximum likelihood practical range (red dashed line).

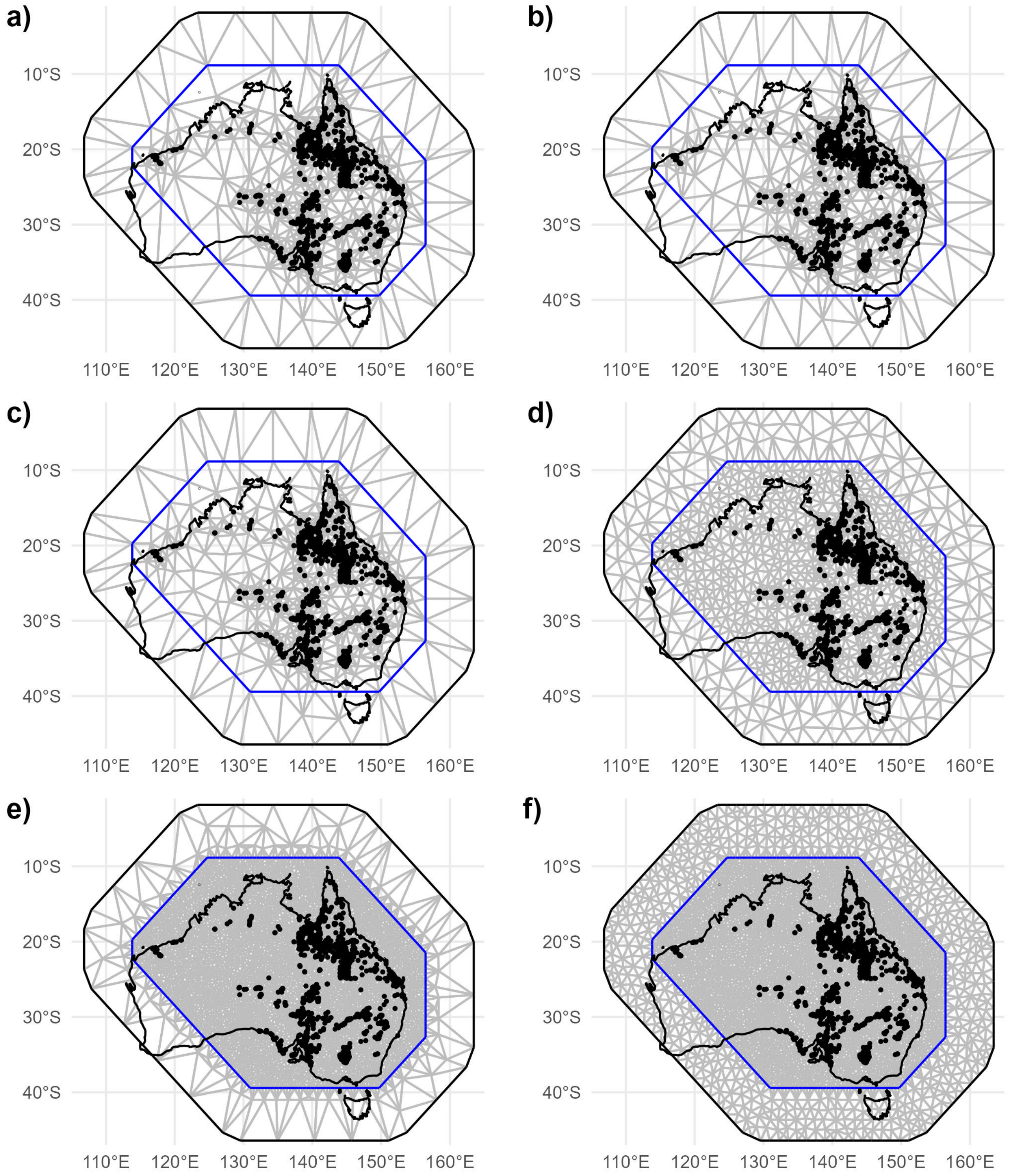

Supplementary Figure 8. INLA-SPDE spatial mesh configurations using maximum triangulation size arguments a) 10,10, b) 7,10, c) 5,10, d) 2,4, e) 1,10, and f) 1,2. The first value in the argument pair is the maximum triangle size in the inner domain and the second value is the maximum triangle size in the outer domain.
